# Role of the interaction between troponin T and AMP deaminase by zinc bridge in modulating muscle contraction and ammonia production

**DOI:** 10.1007/s11010-023-04763-7

**Published:** 2023-05-15

**Authors:** Francesca Ronca, Antonio Raggi

**Affiliations:** Laboratory of Biochemistry, Department of Pathology, University of Pisa, Via Roma 55, 56126 Pisa, Italy

**Keywords:** AMP deaminase, Fast skeletal muscle, Troponin T, Calpain, Zinc-binding motif

## Abstract

The N-terminal region of troponin T (TnT) does not bind any protein of the contractile machinery and the role of its hypervariability remains uncertain. In this review we report the evidence of the interaction between TnT and AMP deaminase (AMPD), a regulated zinc enzyme localized on the myofibril. In periods of intense muscular activity, a decrease in the ATP/ADP ratio, together with a decrease in the tissue pH, is the stimulus for the activation of the enzyme that deaminating AMP to IMP and NH_3_ displaces the myokinase reaction towards the formation of ATP. In skeletal muscle subjected to strong tetanic contractions, a calpain-like proteolytic activity produces the removal in vivo of a 97-residue N-terminal fragment from the enzyme that becomes desensitized towards the inhibition by ATP, leading to an unrestrained production of NH_3_. When a 95-residue N-terminal fragment is removed from AMPD by trypsin, simulating in vitro the calpain action, rabbit fast TnT or its phosphorylated 50-residue N-terminal peptide binds AMPD restoring the inhibition by ATP. Taking in consideration that the N-terminus of TnT expressed in human as well as rabbit white muscle contains a zinc-binding motif, we suggest that TnT might mimic the regulatory action of the inhibitory N-terminal domain of AMPD due to the presence of a zinc ion connecting the N-terminal and C-terminal regions of the enzyme, indicating that the two proteins might physiologically associate to modulate muscle contraction and ammonia production in fast-twitching muscle under strenuous conditions.

## Introduction

Troponin (Tn) is composed by three proteins, troponin I (TnI), troponin C (TnC) and troponin T (TnT), and this complex binds to tropomyosin (Tm) in the thin filament and regulates the contractile activity of striated myofibrils. When Ca^2+^ binds to TnC, the TnI inhibition on the actomyosin ATPase is released exposing the myosin-binding sites on F-actin subunits and allowing cross-bridge cycling and fiber contraction [1].

It is well established that the highly conserved C-terminal domains of fast, slow and cardiac TnT have a role in anchoring the Tn complex to Tm on the thin filament [2, 3]. On the contrary, the N-terminal region has a high degree of variability depending on the muscle type and development stage and no clear function has been envisaged [4–7]. However, it has been reported that the TnT-binding affinity to Tm depends on the sequence and charge of the TnT N-terminal domain [8] further strengthening the hypothesis that TnT might act as a modulator of Tn Ca^2+^ sensitivity and force production [9] as TnT has been found to activate the actomyosin Mg ATPase in the presence of Tm, but without the other components of the troponin complex [10].

Although a protein that binds in vivo the TnT N-terminal elongated region has not been identified yet, it has been reported that the N-terminal domain of rabbit fast-twitch TnT interacts in vitro with rabbit white muscle AMP deaminase (AMPD) [11]. We have also reported that, following the cleavage of its N-terminal region by a calpain-like protease, rabbit skeletal muscle AMPD is no longer inhibited by ATP and that the increase in its catalytic activity might result in the large muscle ammonia accumulation observed during muscle strenuous exercise [12] and that the inhibition of AMPD by ATP lost by limited proteolysis of the enzyme with trypsin that has similar consequences to those caused by calpain in vivo is restored by adding either purified TnT or its phosphorylated N-terminal peptide [11]. We have also reported that the N-terminal domain of rabbit fast TnT might bind a metal ion by a putative zinc-binding motif [13] and that the N-terminal region of rabbit skeletal muscle AMPD contains a putative zinc-binding site that has a critical role in modulating the enzyme activity by fixing the conformation of the dinuclear catalytic zinc center [14, 15]. These observations led us to hypothesize that the interaction of the TnT N-terminal putative zinc-binding site with the catalytic Zn ion present at the AMPD C-terminus simulates the binding of the enzyme N-terminal and C-terminal regions via the dinuclear Zn center reverting the highly active proteolyzed AMPD form to the less active native conformation. The TnT N-terminal region might therefore have a physiological role in switching off the activated AMPD and allowing recycling of IMP and elimination of ammonia after intense muscle contraction [13].

The hypothesis that the modulation of AMPD in fast skeletal muscle might be physiologically regulated by the interaction with the specific N-terminus of fast TnT is strengthened by the observations reported in this review showing that the variability of the N-terminal segment of TnTs and the expression of different TnT isoforms in muscle tissues, that diverge for function and metabolism, are linked to the co-expression of different isoforms of AMPD. A distinctive relationship between TnT and AMPD is further represented by the isoform switch during muscle development, the adaptation to different contractile necessities or the readjusting to the change of environmental conditions, the restricted proteolysis by calpains of the N-terminal domains containing both a putative zinc-binding site and the nuclear localization shared by both proteins.

### Troponin T and the calcium sensitivity of the MgATPase of actomyosin

Contraction of cardiac and skeletal muscle is regulated via the actin filament by tropomyosin (Tm) and the troponin (Tn) complex, composed by TnI, TnC and TnT. It is generally accepted that the calcium sensitivity of the MgATPase of actomyosin is regulated by the Tn complex. TnI is the component of the Tn complex that inhibits the MgATPase of actomyosin by the blocking of the interaction of actin with myosin [16]. As calcium is released, TnI switches to the non-inhibitory mode and the MgATPase is activated. Several results indicate that the switch of the troponin complex on the thin filament is promoted by TnT, the Tm-binding subunit of the Tn complex, that presumably lowers the affinity of TnC for TnI, so removing the inhibition on the MgATPase [17, 18].

**Fig. 1:**
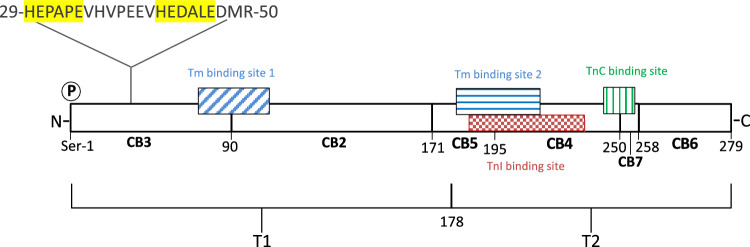
Schematic representation of the primary structure and the functional domains of the 279 aa long fast Troponin T from rabbit white skeletal muscle (Accession number P02641.4). The positions of the CNBr peptides (CB) are indicated [21, 22]. T1 and T2 are the chymotryptic fragments of rabbit fast TnT [19] that contain the Tm-binding sites 1 (79–117 region, blue diagonally striped box) and 2 (179–218 region, blue horizontally striped box) [20], respectively. The region 244–257 (green vertically striped box) corresponds to the human cardiac TnC-binding site [23] and the region 190–235 (red checkered box) corresponds to the chicken fast skeletal muscle TnI-binding site [24] after alignment with the rabbit fast TnT sequence using the Clustal Omega program available at https://www.ebi.ac.uk/Tools/msa/clustalo/. The 29–50 region corresponds to the putative metal-binding site at the N-terminal region of rabbit fast TnT; in yellow are highlighted the two sequences HEPAPE and HEDALE that correspond to two putative Zn-binding sites HEXXXE. **P** indicates that the amino acid residue Ser-1 can be phosphorylated by phosphorylase b kinase and TnT kinase [25, 26]

The two Tm-binding sites that have been identified on striated muscle TnT are localized, respectively, in the T1 and T2 chymotryptic fragments of rabbit fast TnT [19], the first Tm-binding site (T1 fragment) encompassing the aa residues 79–117 of the longest rabbit fast skeletal muscle TnT variant and the second Tm-binding site (T2 fragment) encompassing the aa residues 179–218 [20] (Fig. 1). Even though the aa sequences of the two Tm-binding sites of fast, slow and cardiac TnT isoforms are conserved in avian and mammalian species, the differences in binding to Tm of the TnT isoforms have been attributed to the hypervariability of the N-terminal region of the different TnT isoforms whose expression is regulated during development and in different muscle types by alternative splicing [20, 27, 28].

Although several authors reported that the deletion of the N-terminal region does not eliminate the TnT activity in the Ca^2+^ activation of actomyosin ATPase suggesting that the N-terminal variable region might not be essential in the regulation of muscle contraction [29, 30], others reported that after removal of the N-terminal 38 residues of bovine cardiac TnT (TnT_39–284_) the calcium sensitivity of acto-S1 ATPase decreased [31] and that the fragment TnT_1–191_ of chicken skeletal muscle promoted the same level of activation of actomyosin MgATPase in the presence of Tm acted by the whole Tn complex in the presence of calcium [10]. The unusually long and charged N-terminal region of the chicken breast muscle T1 fragment of TnT showed a lower affinity to a Tm column compared to the recombinant T1 fragment of rabbit fast TnT, while no differences in affinity for Tm were observed for the C-terminal Tm-binding site within the T2 fragment of fast TnT from the two species. The deletion of the N-terminus of the T1 fragment abolishes the differences among fast, slow and cardiac T1 recombinant fragments in the binding affinity for Tm. These last observations might suggest that the hypervariable N-terminus region might exerts a modulatory role on reducing the affinity for Tm by inducing secondary conformational changes in other domains of TnT [8].

It has been established that TnC and TnI are bound at the C-terminal region of TnT [32, 33]. The binding site of TnI was mapped to the C-terminal region of chicken fast muscle TnT (aa 216–263) using deletion mutants [10].The crystallographic structure of a portion of the TnT C-terminal region in the Tn complex confirmed that the TnI-binding site is localized in the C-terminal region of chicken fast skeletal muscle TnT (aa 200–245, corresponding to aa 190–235 in rabbit fast skeletal muscle TnT) [24], and the binding site of TnC to the 256–270 region of human cardiac TnT (corresponding to aa 244–257 in rabbit fast skeletal muscle TnT) [23] (Fig. 1). However, due to its hypervariable sequence, the N-terminal region has different length and acidity in the various TnT isoforms [2] and might also contain sites of contact for TnC [25, 33] and for TnI [34]. Moreover, it has been shown that the presence of an acidic or a basic chicken fast muscle T1 region in chimera proteins formed with the mouse cardiac T2 region modified the binding to Tm and TnI, corroborating the hypothesis that the TnT N-terminus region might modulate the regulatory role of TnT in the fiber contractility [35]. Interestingly, the strongly acidic N-terminal domain of TnT appears to have an extended conformation that might be accessible to phosphorylation by TnT kinase and by phosphorylase b kinase at Ser-1 [25, 26].

### Different TnT isoforms in different muscle fibers

Three different TnT isoforms are encoded from three homologous genes in mammalian and avian cardiac muscle (*TNNT2*), slow skeletal muscle (*TNNT1*) and fast skeletal muscle (*TNNT3*) [6, 36–40] and their expression is tightly regulated during the development of each muscle type.

The three TnT isoforms differ mainly at the N-terminal region [27, 41–43], suggesting that N-terminal variable region might have diverse modulatory functions in different types of muscle and at different stages of development, whereas the C-terminal region, where the core activity of TnT in regulating muscle contraction by binding to TnI and TnC has been located, is highly conserved in the vertebrate TnT isoforms.

The comparison of the TnT sequences showed that the TnT isoforms of the same species are less conserved among the three muscle fiber types than across vertebrates [42, 44], suggesting that the N-terminal TnT structural diversities might be linked to the adaptation to different function, metabolism and contraction mechanisms of cardiac, fast, and slow muscle fibers rather than to species evolution.

The hypothesis that the TnT isoforms have diverse modulatory roles in the different types of muscle fiber is well supported by the example of the heart of the toad *Bufo*. In the toad’s cardiac fibers, TnT1 replaces TnT2 while all other isoforms of the myofilament proteins are expressed as cardiac isoforms [45]. The toad heart was found to be more tolerant to afterload and it exhibited systolic and diastolic velocities faster than the *Rana* frog heart, suggesting that the expression of the slow skeletal muscle TnT isoform might be an adaptation strategy to overcome heat changes and large variations in the volume of body fluid and blood between rainy and dry seasons [45].

Several mutations in *TNNT1*, *TNNT2*, and *TNNT3* genes have been reported to cause cardiac and skeletal myopathies. TnT2 containing an abnormal splicing in the N-terminal region has been linked to cardiomyopathies such as inherited dilated cardiomyopathy in turkey hearts expressing a TnT2 missing exon 8 [46]. Similarly, in dog hearts with dilated cardiomyopathy TnT2 does not contain the constitutively expressed exon 7 [27, 42, 47]. Expression of TnT2 lacking exon 4 was detected in failing human hearts [48, 49], diabetic rat hearts [50] and hypertrophic rat hearts [51]. It has also been suggested that the high incidence of inherited cardiomyopathy and heart failure reported in cats and Guinea pigs could be linked to abnormal splicing of the exons encoding for the N-terminal region of TnT2 [47, 52, 53]. In contrast to the numerous mutations found in cardiac TnT2 gene, fewer mutations in TnT3 gene have been reported in skeletal muscle diseases.

The three TnT isoforms appear to play a nonredundant role in muscle fiber types as evidenced by the two observations that the severe nemaline myopathy (NEM, characterized by the presence of rod-shaped or “nemaline” inclusions in skeletal muscle fibers) with infantile death has been linked to a nonsense mutation in human *TNNT1* gene that truncates the protein at amino acid 180 deleting the binding sites for TnI, TnC and tropomyosin [54, 55] and that the knock-down of the expression of TnT1 resulted in atrophy, slow-to-fat fiber type switch and reduced resistance to fatigue in the mouse diaphragm muscle [56].

Several mutations in the gene encoding TnT1 have been linked to some NEM. A first nonsense mutation was identified in the Amish NEM (MEN5) and other 6 truncation or internal deletion mutations have been reported in other ethnic groups with myopathies like that of MEN5. The marked clinical variability, ranging from neonatal lethal to mild non-progressive forms, is thought to be linked to the developmental regulation of the TnT isoforms in skeletal muscles. During the embryological development, immature TnT isoforms (embryonic TnT3 and cardiac TnT2) are expressed in skeletal muscle. Around birth and progressing through the first months of life, these are replaced by the adult TnT3 and TnT1 isoforms in fast and slow fibers, respectively. The increase in muscle weakness and atrophy in NEM parallels the time course that the mature TnT1 expression would follow, suggesting that the human muscle viability strictly depends on the expression of normal TnT1 in slow fibers [57].

Recently, one case of severe congenital NEM was linked to the deficient expression of TnT3 resulting in the atrophy the type-2 fibers. A spliced variant of TnT3 was found expressed in other two cases of congenital NEM. While respiratory weakness persisted in those patients with TnT3-related congenital myopathy, limb strength improved over time, suggesting that a postnatal adaptation, such as hypertrophy of slow fibers, might have occurred in certain muscle groups [58, 59].

### TnT N-terminal alternative splicing and development

For each of the three muscle-specific TnT genes, alternative RNA splicing during embryonic and postnatal development of skeletal muscle regulates the expression of several isoforms of TnT that differ in the length and acidity of the N-terminal domain [2, 27, 42].

The mammalian cardiac TnT gene is composed by 14 constitutively expressed exons and 3 alternatively spliced exons, two of which encode segments in the N-terminal variable region. The avian cardiac TnT gene contains 16 constitutively spliced exons and 1 N-terminal alternative exon expressed only in embryonic cardiac TnT [37]. In normal cardiac muscle, TnT N-terminal alternative splicing generated 4 and 2 variants in mammals and birds, respectively (reviewed in [7]).

Mammalian fast skeletal muscle TnT gene contains 19 exons. The alternative exons are exons 4, 5, 6, 7, 8 and a fetal exon, all encoding segments in the N-terminal variable region. However, only 13 mouse fast TnT isoforms and 11 chicken fast TnT isoforms differing in the N-terminal variable region are expressed at detectable levels [60–62]. In addition to the alternative spliced exons above mentioned, in the fast TnT gene of the avian orders of Galliformes and Craciformes are also present seven P exons, that express seven tandem repeats of a pentapeptide (AHH[A/E]E) at the N-terminus of the protein forming the so-called Tx segment [61, 63, 64]. Although the physiologic function of this Tx segment is not clear yet, it has been hypothesized that it forms a cluster of high-affinity transition metal ion binding sites [65].

The mammalian slow skeletal muscle TnT gene is composed by 13 constitutive exons and one alternatively spliced exon. Alternative splicing of exon 5 in the N-terminal region generates 2 variants of slow TnT [40, 44, 66]. Splicing at two alternative acceptor sites of intron 5 (in mouse) or intron 4 (in chicken) produces an additional single amino acid variant in the slow TnT isoforms [39, 40].

The significance of the N-terminal alternatively spliced TnT variants has yet to be determined. It has been reported that the presence of a more negatively charged TnT N-terminus conferred to isolated fibers from chicken skeletal white muscles a higher myofilament calcium sensitivity [67–69]. When reconstituted into skinned cardiac muscle strips, the embryonic cardiac TnT with a more negative N-terminus also confers a higher Ca^2+^ sensitivity to myosin ATPase and force development in comparison to the adult cardiac TnT with a less negative N-terminal domain [3]. Moreover, the embryonic cardiac TnT conferred high tolerance to acidosis to embryonic and neonatal cardiac muscle [70]. In contrast, the tolerance to acidosis was decreased in transgenic mouse cardiac muscle when fast skeletal muscle TnT with a less negatively charged N-terminus was overexpressed [71].

Whilst cardiac TnT is expressed at significant levels during the embryonic development of avian and mammalian skeletal muscles, during postnatal development a switch from the cardiac to the skeletal muscle TnT isoforms is observed (reviewed in [7]).

During the development of mammalian skeletal muscles, the N-terminal variable region of fast skeletal muscle TnT is subjected to a complex alternative splicing and the inclusion of the fetal and of the other N-terminal alternative exons (exons 4, 6, 7, and 8) decreases as adulthood is reached resulting in a developmental switching from high molecular weight acidic isoforms to low molecular weight basic isoforms [27, 43, 62, 72].

Interestingly, the seven P exons encoding the Tx segment in the N-terminal region is expressed post-hatch only in the adult fast TnT isoforms of some birds [60, 63]. It has been suggested that the high number of negative charges of the Glu residues of the Tx domain might have a role in binding divalent metal ions and that it could act as a calcium reservoir [73]. The N-terminal Tx segment of avian fast TnT, composed by a transition metal-binding histidine-rich sequence HE/AEAH repeated four to seven times, was found to have great affinity for zinc. Since the unique Tx segment of fast TnT is expressed in the fast glycolytic fibers of the breast pectoralis muscle but not in leg muscles of Galliformes [65, 74], a link might exist between the presence of the Tx sequence and the fly capacity of these birds that despite their large flight muscles show little endurance to long fly but a rapid burst fly pattern. Structural changes induced by Zn^2+^ binding to the Tx segment of chicken breast TnT appeared to alter the binding affinity of TnT to Tm [75]. In addition, the histidine-imidazole groups of the Tx region might change protonation- deprotonation state when cellular pH drops during anaerobic work, possibly modifying not only the conformation of the N-terminal domain of TnT [76] but also the binding of fast TnT to Tm that has been shown to be stabilized by lower pH [60]. The hypothesis that the TnT N-terminus might bind divalent transition metals rather than calcium is strengthened by the observation that in rabbit fast skeletal TnT the N-terminal region contains the sequence of residues 29–50 (^29^HEPAPEVHVPEEVHEDALEDMR^50^) where two putative zinc-binding sites (HEXXXE) are located, as it has been ascertained that the similar HEXXXH motif in the N-terminal region of rabbit muscle AMP deaminase represents a zinc-binding site [14]. This observation applies also to the human fast skeletal TnT that reveals the presence of a similar zinc-binding segment (HEEVHE).

### Posttranslational modifications of the TnT isoforms

TnT undergoes posttranslational modifications such as phosphorylation and restricted proteolysis at the N-terminus of the protein. Recent mass spectrometry data showed that under physiological conditions the totality of rat adult cardiac TnT is phosphorylated at Ser1 [77, 78] confirming a previous report by Perry [2]. Furthermore, the embryonic mouse cardiac TnT, overexpressed in adult heart, was found to be fully phosphorylated at Ser25 [79], suggesting that the phosphorylation at the N-terminus is conserved in the different isoforms during development and might have an important regulatory role.

In pathological conditions, such as acute ischemia–reperfusion or pressure overload, the phosphorylated 71 amino acid N-terminal variable region of cardiac TnT undergoes a restrictive proteolysis by calpains. The selective removal of the N-terminal domain does not abolish the function of cardiac TnT but alters the binding affinities for TnI, TnC, and tropomyosin and lowers the myosin ATPase activity and myofibril force generation [80, 81] without affecting thin filament calcium sensitivity [82–84].

An intriguing and novel role for fast skeletal muscle TnT has been reported by Zhang et al. [85]. Mouse full-length fast skeletal muscle TnT3 was found to shuttle to the nucleus predominantly in the young muscles, whilst its C-terminal fragment predominated in the old fibers. Decreased full-length fast TnT and increased C-terminal domain of fast TnT in the nucleus impaired the expression of the voltage sensor Ca^2+^ channel α1 subunit reducing muscle force in aging sedentary mice. A calpain inhibitor prevented TnT3 fragmentation and Ca^2+^ channel downregulation improving muscle force generation in sedentary old mice. Whether TnT3 plays a novel transcription role is still investigated but the authors have suggested that the proteolysis of TnT and the nuclear translocation of fast TnT C-terminal fragment might contribute to the development of age-related sarcopenia [86].

Whilst it is well accepted that the N-terminal variable region of TnT does not contain binding sites for the other components of the troponin complex or for tropomyosin, the significance of its role in the contractile fiber has not been elucidated yet. The questions on the functions of its variability, on the possible interactions with other proteins and/or with divalent cations and on the role of its proteolysis during stress events remain unanswered.

Although no protein has been reported to specifically bind the N-terminal domain of TnT, we have previously demonstrated that the rabbit fast skeletal TnT or its phosphorylated 50-residue N-terminal peptide could restores the allosteric properties of rabbit skeletal muscle AMPD removed by limited proteolysis by trypsin in vitro as well as by a calpain-like proteinase in vivo [11, 12]. Based on our findings we have proposed a model where during strenuous exercise the regulatory site represented by the Zn-binding N-terminal domain of the AMPD1 catalytic subunit is released by calpain and the enzyme shows an unrestrained activity producing large quantities of ammonia. In those conditions, the N-terminal domain of TnT through the Zn ion contained in its putative zinc-binding motif might bind the remaining catalytic Zn ion of the cleaved AMPD1 restoring the allosteric properties of the enzyme through the formation of a vicarious dinuclear Zn metallocenter [13].

### Functional characteristics of AMPD isoforms in muscle fibers. The nature of AMPD1 as zinc metalloenzyme

The hydrolytic deamination of AMP to IMP and NH_3_ is catalysed by AMP deaminase (AMPD), a zinc enzyme that, although is distributed in most animal tissues, shows a particularly elevated activity in skeletal muscle [87].

Extensive work carried out by Parnas and his school well established that ammonia formed by muscle during work arises from the reaction catalyzed by AMPD. Parnas considered irreversible the deamination processing of AMP, which occurs during anaerobic contraction, and hypothesized the existence of a regenerative process of AMP from IMP occurring during periods of oxidative recovery [88].

Parnas ruled out that the ammonia production was directly linked to muscle contraction and proposed that the AMP deamination to IMP was instead the result of the low efficiency of anaerobic glycolysis to convert the AMP produced during muscle contraction into ATP [89]. According to Parnas, the absence of a direct correlation between the NH_3_ production and muscle contraction was in accord with the observation that NH_3_ increase was not proportional to the muscle work but it varied based on the duration of the muscle stimulation and on the physiological condition of the animal [90].

During development from fetal to adult muscles, Kendrick-Jones and Perry [91] observed a transition for AMPD similar to that reported for other metabolic enzymes such as adenylate kinase (AK), creatine phosphokinase (CK) and fructose 1,6-diphosphate aldolase, whose activities increase in skeletal muscle development well in accord with the observation that the adult skeletal muscle showed a more highly developed anaerobic metabolism than its fetal counterpart [92].

AMPD is present as multiple isoforms in muscle fibers. Three different AMPD isoforms are sequentially expressed during the development in rat muscles. The embryonal muscles express the AMPD isoform B that is specific of adult non-muscle tissues such as spleen, lung, thymus, blood and liver. Near birth and for the first 2–3 week of life, the perinatal AMPD isoform, corresponding to the heart isoform C, is encoded in the perinatal skeletal muscles together with the adult skeletal muscle AMPD isoform that reaches its maximum expression after 3 weeks of development [93]. Interestingly, as discussed in the above paragraph for the N-terminus of fast skeletal TnT [62], changes in the isoform expression during muscle development, with a timeline similar to the AMPD one, have been reported also for the main contractile proteins, i.e., myosin heavy and light chains, actin, tropomyosin, troponin [93], suggesting that the expression of different isoforms of those proteins may be coordinated during myocyte development.

Three mammalian genes encode for the different muscle isoenzymes, i.e., *ampd1*, highly expressed in skeletal muscle, and *ampd2* and *ampd3* that express the smooth and cardiac muscle isoforms, respectively [94]. The N-terminal domains of AMPD1, AMPD2 and AMPD3 isoenzymes are highly divergent while the C-terminal domains are highly conserved. It has also been shown that striated muscle contains two different isoenzymes of AMPD [95–97]. The red muscle main form (form A) corresponds chromatographically to the only isoenzyme present in the cardiac muscle (AMPD3), while the minor component found in red muscle (form B) corresponds to the white muscle isoenzyme AMPD1, also called form M [96].

At the optimal pH value 6.5, both isoenzymes show hyperbolic substrate–velocity curves that are reverted to sigmoid by the inhibitory effect of GTP. Form A is almost insensitive to ATP whilst this nucleotide, likewise to GTP, exerts on form B an inhibitory effect. Interestingly, the modulation of the two isoforms differs at pH 7.1 that can be assumed to be the resting fiber pH. At pH 7.1 both enzymes follow sigmoid kinetics, but ATP inhibits form B whilst it stimulates form A, reverting sigmoidal to hyperbolic kinetics shown by the enzyme at optimal pH [97]. The kinetic properties of AMPD isoform M isolated from rabbit skeletal muscles that are mainly composed of white fibers are similar to those of isoform B of red muscle. At physiological concentration of AMP (0.1 mM) isoform M is strongly inhibited by ATP at pH 7.1 whilst ADP modulates the enzyme to follow hyperbolic kinetics even in the presence of ATP. These data could be interpreted as that AMPD is inhibited by ATP during resting or moderate contractile activity of white skeletal muscle, whilst it is activated during strenuous anaerobic contraction, when a decrease in ATP and an increase of ADP levels occur together with the acidification of tissue due to the production of lactate [98].

It is well accepted that the role of AMPD1 is to regulate the relative concentration of adenine nucleotides during muscle contraction: AMPD1 removes AMP from the equilibrium of the AK reaction 2 ADP= ATP + AMP enhancing the production of ATP for the myosin ATPase [13]. The observation that, in contrast with the other AMPD isoforms, including the cardiac one [95], that are activated by ATP, AMPD1 is inhibited by ATP and activated by ADP well supported the hypothesis that the increase in activity of AMPD1 during intense muscular activity is due to the decrease in the ATP/ADP ratio [98].

We reported that the catalytic subunit of rabbit white muscle AMPD1 contains a structurally bridged dinuclear metallocenter [15] composed by the catalytic zinc bound to the C-terminus of the enzyme (corresponding to the single zinc bound at the catalytic site described for murine adenosine deaminase [99] and yeast AMPD [100]) and a zinc bound to the putative zinc-binding site in the N-terminal region. The rabbit histidine-proline-rich-glycoprotein (HPRG) has been reported to be associated with the catalytic subunit of rabbit white skeletal muscle AMPD1 [101] to form a 1:1 molecular adduct (two 85 kDa catalytic subunits assembled with two 73 kDa HPRG subunits) [102–104]. When the HPRG component was separated by zinc-affinity chromatography the solubility of the catalytic subunit of the enzyme was markedly reduced [105] strengthening the hypothesis that HPRG functions as a zinc metallochaperone in the formation of the AMPD1 heterotetramer. It was also reported that HPRG is produced only by the liver [106] and that the rabbit skeletal muscle cells internalize the serum protein [107]. Our data have been confirmed by recent in vivo experiments [108] that showed that HPRG, after intravenous injection, was internalized quickly in healthy tissues, including muscle, and tumors.

### Functional implications of the presence of a highly differentiated N-terminal region in the AMPD1 catalytic subunit

The removal by trypsin of the 95 amino acids long N-terminal fragment of the rabbit AMPD1 isoform M induces a change in the regulatory properties of the enzyme that becomes more active at low substrate concentration and is no longer inhibited by ATP at optimal pH 6.5 [109–111]. Interestingly, a 97 amino acids long N-terminal fragment could be released in vitro from rabbit AMPD1 by a protease with a specificity identical to that reported for ubiquitous calpains [12, 112] and it had been also reported that during storage the human AMPD1 and AMPD3 recombinant isoenzymes were cleaved to mixtures of two polypeptides truncated at the N-terminal region (△I86–△H98 and △L88–△M90, respectively) [113]. All cleavage sites have a Leu at P2, this specifity suggesting that the proteolysis might be mediated by a calpain protease, and that this phenomenon might also occur in vivo playing a role in modulating the AMPD isoenzymes in striated muscle [12]. Interestingly, the N-terminus of rabbit AMPD1 contains a sequence (KELLDA) that is also present in the calpastatin inhibitory domain [114] that might block the access of calpain to its cleavage site during resting or normoxic contractions, thereby protecting against the protease-induced fragmentation of AMPD1 [12]. On the contrary, a strong tetanic contraction in skeletal muscle might induce the activation of a calpain resulting in a large ammonia accumulation due to the increased activity of AMPD that deaminates AMP to IMP.

**Fig. 2:**
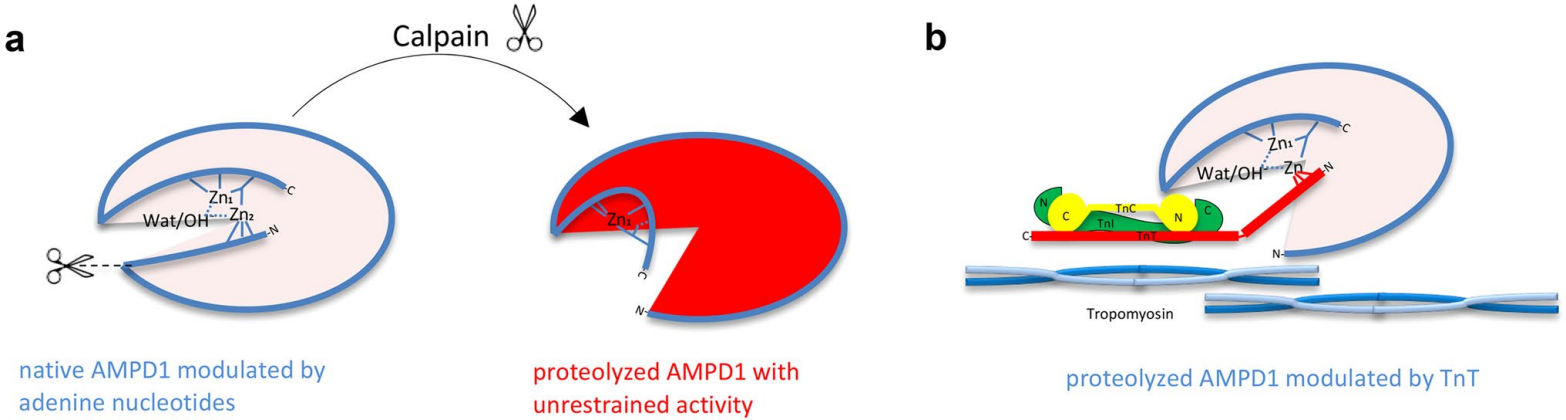
Modulation of the calpain-proteolyzed rabbit AMPD1 by the N-terminal Zn-binding region of rabbit fast TnT. **a** The native AMPD1 catalytic subunit, in pink, is allosterically inhibited by ATP and activated by AMP and ADP. The scissors indicate the in vivo proteolytic activity of calpain on AMPD1. Proteolyzed AMPD1, in red, shows an unrestrained activity following hyperbolic kinetics, no longer modulated by adenine nucleotides. The coordination of the zinc ion in proteolyzed AMPD is based on the model proposed for the catalytic site of adenosine deaminase [99]. **b** Model of tropomyosin and troponin complex with the extended N-terminal domain of the TnT protein depicted according to Amarasinghe and Jin [8]. The TnT N-terminal domain inhibits the activity of proteolyzed AMPD1, restoring the allosteric properties of the native enzyme, i.e., inhibition by ATP and activation by ADP

Our hypothesis that the unrestrained increase in AMPD1 activity upon proteolytic cleavage during strenuous exercise could be reverted by the binding to AMPD1 of the phosphorylated N-terminal region of TnT is supported by our previous data that rabbit fast TnT or its phosphorylated 50-aa residue N-terminal peptide restores in rabbit AMPD1 the inhibition by ATP, removed in vitro by limited proteolysis by trypsin causing the release from the enzyme of a 95 amino acids N-terminal fragment [11]. Since the N-terminal region of fast rabbit TnT contains a putative zinc-binding motif, it could mimic the regulatory action of the AMPD1 N-terminal domain that, when present, forms a dinuclear zinc site with the C-terminal catalytic zinc and maintains the enzyme in its native conformation showing both the positive homotropic behaviour (i.e., activation by the substrate AMP) and the modulation by adenosine di- and triphosphates (i.e., activation by ADP and inhibition by ATP) (Fig. 2).

Our recent study confirmed the presence of a dinuclear zinc site in rabbit AMPD1 showing that the carbethoxylation of His-51 of the Zn_2_ coordination sphere removes the inhibition by ATP at pH 7.1 through a conformational change of the enzyme C-terminal region, where an ATP-binding site has been localized [115].

### Localization of AMPD1 and histidine-proline-rich-glycoprotein (HPRG) in the skeletal muscle fiber

Based on the early observations, AMPD has been found to be associated with various proteins of the contractile machinery [116]. The increase in AMPD activity appeared to be the result of a reversible interaction between AMPD and myofibrils promoted in vivo by intense muscle contraction, suggesting that the role of the enzyme is linked to the maintenance of the adenylate charge under conditions of intense ATP consumption [117–119]. Several data on AMPD1 localization in skeletal muscle were obtained by immunostaining of both isolated myofibrils and muscle fibers grown in culture. In unstretched chicken and human muscle myofibrils, AMPD1 staining was located at the lateral ends of the A-band extending into the I-band [120, 121] and could also be found in the center of the A-band, in correspondence to the M-line [120] where other enzymes such as creatine kinase and adenylate kinase have been localized [122]. In agreement with those data, in rabbit fast-twitch muscles the AMPD1 immunostaining was predominantly located in the A-band in a region significantly beyond the end of the myosin filament. It has also been reported that AMPD1 may be attached to the myofibrils through titin molecules, although the interaction of the enzyme with either titin or myosin had no clear influence on its activity [123, 124].

AMPD immunohistochemistry in human skeletal muscle indicated that the three main isoforms are localized in different parts of muscle tissues [120]. AMPD isoform M (AMPD1) was localized predominantly in Type II (fast-twitch) fibers, mainly concentrated subsarcolemmally and intermyofibrillarly and arranged in a cross-striation pattern. Interestingly, AMPD1 was also concentrated at the neuromuscular junctions co-localizing with acetylcholine receptor clusters and in capillary endothelial cells. AMPD isoform L (AMPD2) was localized mainly in myelinated axons and presynaptic areas and, in a moderate concentration, in smooth muscle cells and endothelial cells. No localization was detected in muscle fibers. AMPD isoform E was localized mainly in smooth muscle cells, endothelial cells, and, in a lower concentration, in nerve bundles. In skeletal muscles, AMPD E was detected mainly in Type I (slow-twitch) fibers with a cross-striation pattern similar to AMPD isoform M.

Histidine-proline-rich-glycoprotein (HPRG) is an approximately 70 kDa glycoprotein that has been isolated from the plasma of several mammalian species, but is also found in infant urine, colostrums, milk, platelets, megakaryocites and immune cells such as monocytes and macrophages (reviewed in [125]). HPRG was found to associate with rabbit skeletal muscle AMPD [101]. An investigation by x-ray absorption spectroscopy revealed that the isolated HPRG component of rabbit AMPD1 might contain two Zn ions, coordinated by His and Cys residues [102]. A zinc-binding site that might coordinate the Zn ion with an amino acid arrangement compatible with the histidine-cysteine-containing site of rabbit HPRG appears to be conserved in several mammalian species [103].

Interesting results were found on the localization of HPRG in skeletal muscle. An anti-HPRG antibody selectively marked the type IIB fibers that contain the highest level of AMPD isoform M [126]. Furthermore, a clear correlation between the muscle HPRG content and the level of AMPD activity was obtained from the immunological analyses of human skeletal muscle biopsies from patients with AMPD deficiency [127]. Our immunohistochemical study on human gastrocnemius and quadriceps femoris has localized HPRG preferentially at the I-band level [128]. Interestingly, we also found a nuclear localization of HPRG in the human skeletal muscle, similarly to what was observed with TnT by Zhang *et al.* [85, 86] that reported that fast-twitching skeletal muscle TnT is fragmented in aging mice and both the full-length TnT and the C-terminal domain of TnT can shuttle to the nucleus and regulate the expression of the voltage sensor Ca^2+^ channel α1.

The hypothesis that the HPRG-AMPD1 complex might interact with proteins of the thin filament is strengthened by our preliminary data that following the limited tryptic proteolysis of the isolated HPRG component of rabbit AMPD1 actin fragments are released and can be isolated (Ronca and Raggi unpublished observation). The observation that HPRG binds with high-affinity and in a Zn^2+^ or pH-dependent manner to tropomyosin present on the surface of the FGF-2-activated HUVEC cell [129] strengthens our hypothesis that HPRG acts as a zinc metallochaperone, modulating its intracellular availability for the formation of the dinuclear zinc site critical for AMPD1 stability in its native allosteric conformation [14, 105, 125]. More recently, transmission electron microscopy and immunogold analysis evidenced the presence of HPRG in the sarcomeres, mainly in the I-band. Using the double immunogold staining, a colabeling of HPRG and AMPD1 was evident at sarcomeric, sarcoplasmic reticulum and nuclear levels [130].

### Evolution among mammalians of the role of the fast troponin T phosphorylated N-terminus as Zn-binding region in the modulation of skeletal muscle AMP deaminase

To explain the involvement of the N-terminal Zn-binding regions of rabbit fast AMPD and TnT in the modulation of the enzyme activity, we have proposed the model of interaction between the AMPD1 catalytic subunit and the TnT N-terminal domain which is depicted in Fig. 2 (modified from [13]).

The in vivo proteolytic activity of calpain removes the N-terminal domain of AMPD1 with his putative Zn_2_ binding site involved in the formation of the dinuclear metallocenter, causing the enzyme to show an unrestrained activity that follows hyperbolic kinetics no longer modulated by adenine nucleotides. The interaction of the zinc ion present at the putative zinc-binding site of the TnT N-terminal domain with the catalytic Zn ion (Zn_1_) of the enzyme might reconstitute the zinc dinuclear center that exists in native AMPD through the interaction of the catalytic Zn_1_ ion present at the C-terminus of the enzyme with the putative Zn_2_ ion at the N-terminus at which the competition between activatory (AMP and ADP) and inhibitory (ATP) adenine nucleotides takes place. This model is supported by the experimental observation that the addition in vitro of the phosphorylated N-terminal domain of TnT to AMPD1 proteolyzed by trypsin restored the sigmoid kinetics and the allosteric properties of the native enzyme, i.e., activation by ADP and inhibition by ATP [11].

Whilst the C-terminal region of AMPD1 is thoroughly conserved among vertebrates and always contains the motif SLSTDDP believed to be part of the Zn catalytic center, the N-terminal region differs among the enzyme isoforms.

Data obtained by EXAFS suggested that rabbit skeletal muscle AMPD contains an active site formed by a dinuclear zinc site [14] similarly to other dinuclear zinc hydrolytic enzymes such as dihydroorotase [131] and phosphotriesterase [132]. We provided evidence that the two zinc ions would connect the N-terminal and C-terminal regions of the enzyme being coordinated by 2 histidines and 2 or 3 O ligands (Zn_1_ and Zn_2_, respectively) and bridged by a carboxylate and a water/hydroxyde anion. One of the zinc ions (Zn_1_) is located at the enzyme C-terminus and acts to polarize a nucleophile water molecule, whereas the other (Zn_2_) is probably bound to the region HHEMQAH (residues 51–57) at the enzyme N-terminus and appears to exert a regulatory role and confer the cooperative behaviour of AMPD1 by transiently binding an activating substrate molecule [15].

Based on our findings of a 309-kDa molecular mass for native rabbit AMPD1 by sedimentation–equilibrium centrifugation [110], we have proposed an AMPD heterotetramer composed of two 85-kDa catalytic subunits assembled with two 70-kDa HPRG subunits [105]. In our opinion, the determination by atomic absorption spectroscopy of 2.6 g atoms of Zn per mole of rabbit AMPD1 [133] is consistent with the presence in the AMPD–HPRG complex of a dinuclear zinc site per AMPD catalytic subunit whose N-terminus was presumably proteolyzed, reducing to 278 kDa the value of the extimated enzyme MW. Further data showed that the rabbit AMPD1 apoenzyme binds 4 g atoms of zinc per mol but the increase of V_max_ due to the addition of the fourth zinc atom is only 28% of that expected, suggesting that the fourth zinc atom is not directly associated with the catalytic activity [133].

We have reported that His-51 carbethoxylation by DEP in native rabbit AMPD1 causes a loss of the sensitivity of the enzyme to ATP both at pH 7.1 and at pH 6.5, suggesting that the chemical modification of the Zn_2_ binding site at the N-terminus of the catalytic subunit of rabbit AMPD1 alters the Zn_1_ binding coordination and change the conformation of the C-terminal region of the enzyme where an ATP-binding site has been localized [134]. The trypsin-induced removal of the 95-residue N-terminal region of the rabbit enzyme mimics only in part the effect of the carbethoxylation of His-51. We have reported that the trypsinized enzyme at optimal pH 6.5 exhibits hyperbolic kinetics even at low K^+^ concentration and is no longer inhibited by ATP, but the limited trypsinization does not abolish neither the inhibitory effect by ATP at pH 7.1 nor the pH-dependent stimulation of AMPD1 by substrate [110]. The different effects of carbethoxylation of His-51 and proteolysis of the N-terminal domain of rabbit AMPD1 might be explained with the observation that the proteolysis produces major fragments (residues 24–57, 31–57, 31–72, 36–72), each containing the complete Zn_2_ binding site, that still could concur to form the dinuclear zinc-binding site.

We have reported that purified rat AMPD1 (MW = 290,000) contains 2 g atoms of zinc per mol of enzyme [135]. Rat AMPD1 has always been isolated as a proteolyzed enzyme retaining the inhibition by ATP at pH 6.5 that in the rabbit AMPD1 is lost following proteolysis. No desensitization towards the nucleotide was obtained even with the limited proteolysis of rat AMPD1 by trypsin carried out following the same protocol used with the rabbit enzyme [111], suggesting that rat and rabbit AMPD1 might differ in their regulatory behaviours.

**Fig. 3:**
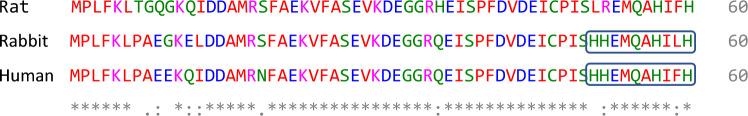
Multiple amino acid sequence alignment of the AMPD1 sequence from different mammalian species. The putative Zn_2_ binding site (HHEMQAHILH and HHEMQAHIFH in the rabbit and human sequences, respectively) is highlighted (square box). Sequences were obtained from the NCBI databank (http://www.ncbi.nlm. nih.gov/). NCBI accession numbers: XP_002715794.3 (rabbit: *Oryctolagus cuniculus*), NP_620231.1 (rat: *Rattus norvegicus*), NP_000027.3 (human: *Homo sapiens*). Default parameters were used for the multiple sequence alignment using the CLUSTAL OMEGA program available at https://www.ebi.ac.uk/Tools/msa/clustalo/. “*” fully conserved residues; “:” conserved substitutions; “.” semi-conserved substitutions

The comparison among human, rat and rabbit AMPD1 reveals that rabbit His-51, His-52, Glu-53 and His-57, involved in the formation of the putative Zn_2_ binding site, are conserved in human but not in rat enzyme (Fig. 3), indicating that the divergence in amino acid sequence localized in residues 51–52 (H–H in the rabbit and human enzymes compared to L-R in the rat), might confer different catalytic and regulatory properties to the different AMPD1 isoenzymes and strongly suggests that the dinuclear Zn site might be present in human AMPD1 conferring to it a specific activity and regulation similar to that of the enzyme expressed in the glycolytic muscle fibers of the rabbit. Given the phylogenetic proximity of rabbit to human [136] the complex regulation of rabbit AMPD1 might have been conserved during evolution and might have contributed to the development of specific roles of fast-twitch muscles in the human behaviour.

In a recent review we have extensively described the data that support the hypothesis that HPRG might act as a zinc chaperone for AMPD1 [104]. In addition to the data of colocalization of AMPD1 and HPRG discussed above, HPRG is consistently purified as a complex with rabbit skeletal muscle AMPD1 and the isolation by zinc-affinity chromatography of the HPRG component resulted in the precipitation of the AMPD1 catalytic subunits [105]. Furthermore, a molar ratio HPRG/AMPD minor than 1:1 causes the inactivation of the enzyme at low AMP concentration [105] in line with the dilution-induced homotropic cooperativity in rabbit AMPD1 [137], suggesting a critical role of HPRG in the maintenance of the native quaternary structure of the enzyme. This view is confirmed by the quaternary structure of wild-type human AMPD1 recombinant protein showing that this enzyme exists as a large instable complex (> 2000 kDa) [113]. In our opinion, the behaviour of the recombinant enzyme might result from the aggregation-induced precipitation of the isolated native 85 kDa catalytic subunit.

Although HPRG has been shown to bind to various soft metal ligands and to regulate many biological activities, a likely role as metallochaperone in the formation of AMPD1 heterodimer with skeletal muscle AMPD is well supported by the experimental data that HPRG isolated from rabbit AMPD1 hosts two Zn^2+^ ions most probably coordinated by His residues and sulfur from cysteine residues [102]. The EXAFS spectrum of the Zn–AMPD–HPRG complexes with various HPRG content demonstrated that no zinc is bound to HPRG when the ternary complex of HPRG with Zn and the AMPD catalytic subunit is formed [14] indicating that HPRG might transfer the metal to the enzyme similarly to what has been documented for the association of superoxide dismutase (SOD1) with its copper chaperone (CCS) that transfers copper to SOD1 probably by forming a temporary intermolecular disulfide bridge [138]. Following the comparison of the HPRG sequence of several placental mammalian species [103], the protein appears to contain, in the so-called PRR1 region, one or two conserved zinc-binding sites formed by at least five amino acid residues known to participate in several soft metal-binding sites (for the rabbit HPRG: Asp-252, His-253, His-257, Cys-264 and Cys-294 that are in agreement with the above mentioned data of the EXAFS analysis of the 2:1 Zn-rabbit HPRG complex). The appearance in the Anthropoidea of a new and highly conserved M-S-C-S/L-S/R-C metal-binding motif, that matches the MxCxxC motif characteristic of metal transporters and metallochaperones, strengthens the hypothesis that HPRG might act as a zinc chaperone of the fast-twitching muscle AMPD1. The high degree of conservation of the HPRG sequences in the Anthropoidea and the conservation of the N-terminal zinc-binding region between rabbit and human AMPD1 might indicate that a functional specialization of the HPRG-AMPD1 tetramer has occurred during evolution that might be associated with the evolution of new white muscle distribution and functions.

Genes that encode the subunits of troponin are found in all invertebrate animals excluding jellyfish and sponges (Cnidaria) and it’s tho

ught to have emerged in parallel with the development of bilateralism and a central nervous system in order to regulate the alternance of muscle contraction and relaxation in coordinated movement of animals [139]. Invertebrate and vertebrate TnT share a conserved core structure and corresponding functions [139]. Similarly to what happens in the vertebrates, several TnT N-terminal variants are generated via alternative RNA splicing of the single invertebrate TnT gene. Interestingly, the TnTs of arthropods and some flying insects such as *Lethocerus* and *Drosophila*, have an extended C-terminus containing 49 glutamic acid residues of unknown function [140–142]. With the hypothesis that the C-terminus Glu-rich domain of *Drosophila* TnT might have a role as a Ca^2+^ buffer/reservoir, mutant flies expressing a C-terminus deletion of TnT have been tested in various muscle exercises. Although the C-terminal deleted TnT maintained its core functions in muscle contraction, the mutant flies had decreased capabilities in both acute and chronic muscle activities, such as climbing velocity, acute flight performance and endurance. Furthermore, the observation that the mutant fly hearts were more resistant to acute Ca^2+^ overloading produced by the electrical pacing–induced membrane depolarization of cardiomyocytes supported the hypothesis that removal of the long Glu-rich C-terminal extension of *Drosophila* TnT diminishes the Ca^2+^ buffering capacity in the myofilaments by lowering the baseline content of Ca^2+^ and allowing the cardiac muscle to better tolerate acute Ca^2+^ overloading [143]. Before these studies in Drosophila, a similar role as a Ca^2+^ buffer/reservoir has been previously proposed for the His and Glu-rich N-terminal domain of the avian pectoral muscle fast TnT expressed in Galliformes and Craciformes [73]. The adult chicken breast muscle TnT was found to bind multiple calcium ions per protein molecule in a non-saturable fashion with affinity in the micromolar range. The atomic model simulations with varying numbers of calcium and zinc ions predicted that both calcium and zinc ions could bind with high-affinity to the N-terminal chicken breast muscle TnT but, consistently with previous data discussed below, only the binding of Zn^2+^ changed the protein conformation. The physiological role of TnT in avian pectoralis muscle could be that of binding Ca^2+^ toward the end of a calcium wave, reducing therefore the twitch length and giving the advantage of a faster contraction cycle in avian flight muscles.

It should be noted, however, that the sequences of the drosophila and chicken cation binding segments differ considerably in amino acid composition, charge and position in the TnT protein: the N-terminal domain of avian fast TnT contains 18 His residues and 24 Glu residues over a 80-aa fragment (^1^MSDTEEVEHGEAHEAEEVHEEAHHEEAHHEEAHHEEAHHAEAHHAEAHHEEAHAHAEEVHEPAPPPEEKPRIKLTAPKIP^80^) whereas the C-terminal domain found in Drosophila contains 50 Glu residues and no His residues over a fragment with the same length (^n^WFGERPGKKAGEPETPEGEEDAKADEDIVEDDEEVEEEVVEEEDEEAEEDEEEEEEEEEEEEEEEEEEEEEEEEEEEEEE^m^). In the avian sequence the His and Glu residues are arranged as a seven-repeated sequence HE/AEAH, designated Tx, that closely resemble a binding site for transition metal ions and has been demonstrated to have high-affinity for Cu^2+^  > Ni^2+^  > Zn^2+^ ≈ Co^2+^ [65]. Similar zinc-binding sequences HEXXH and HEXXXH have been described in various zinc peptidases [144, 145]. Experimentally, it has been demonstrated that both the N-terminus and the full-length chicken breast muscle TnT could be purified by Zn^2+^ affinity chromatography, whereas they were not retained on the Mg^2+^, Ca^2+^, Mn^2+^ or Fe^2+^ columns [65, 73]. Furthermore, the binding of Zn^2+^ to the N-terminal region of chicken breast muscle TnT changed the conformation of the protein and its binding to Tm, TnI, and TnC [146]. In contrast, addition of Zn^2+^ ions had no significant effect on the interaction with Tm of the chicken leg muscle TnT3 isoform that does not contain the Tx sequence [75].

In consideration of the above-described discrepancies of the literature, it would be interesting to investigate whether Zn^2+^ has higher affinity than Ca^2+^ for the N-terminal region of chicken breast muscle TnT and would displace the Ca ion(s) bound to the protein. In addition, it would be worthy to study whether the ability to bind one specific divalent cation is affected by the phosphorylation status of Ser_1_ since we have demonstrated that the phosphorylated 50-residue N-terminal peptide of rabbit fast skeletal TnT restores the allosteric properties of rabbit skeletal muscle AMPD removed by limited proteolysis by trypsin in vitro as well as by a calpain-like proteinase in vivo. Treatment of the peptide with alkaline phosphatase abolishes the modulating properties of the peptide, suggesting that phosphorylation-dephosphorylation processes might be involved in the regulation of the enzyme [11, 12].

The functional significance of the presence of multiple metal-binding sites in the TnT isoform expressed in the fast glycolytic fibers of the pectoralis muscles of Galliformes and Craciformes is not clear yet. It has been suggested that the binding of metal ions to the variable N-terminal region of the Tx-positive TnT isoforms might modify the function of TnT in muscle contraction and could be regarded as an evolutionary adaptation that brought to the development of their rapid burst fly pattern [65]. However, the presence of fast TnT isoforms containing one or two putative Zn-binding sites within the N-terminal region can be detected in many other birds in agreement with previous data that Tx-positive TnT proteins could be also detected by a monoclonal antibody raised against chicken Tx-containing native TnT in the white muscle of several avian species [63]. With increasing depositions of new animal sequences in the NCBI database, the same Zn-binding motif can be found in few of the fast TnT isoforms of jawless fish, cartilaginous fish, bony fish, amphibians and reptiles, suggesting that the putative zinc-binding motif might have appeared early in the evolution of Cordata, whereas, based on the sequences available to date, the putative Zn-binding site of AMPD1 is restricted to the Primata and to the phylogenetically close rabbit. Further studies are needed to determine whether the acquisition of a dinuclear zinc-binding site in AMPD1 in higher Mammalia and the ability to interact with the TnT N-terminal region has a significance in the development of an advanced way to regulate the muscle contraction.

## Conclusions

We report in this review several correlations between AMPD and TnT worth noting. In higher vertebrates three AMPD isoenzymes are differentially expressed in fast skeletal, slow skeletal, and cardiac muscle fibers. Similarly, three homologous TnT genes encode for various TnT isoforms that differ mainly in muscle type expression, length, and charge of the N-terminal region, while the central and C-terminal regions are highly conserved [7]. The ability of the TnT variable N-terminal region to interact with zinc and with other muscle fiber proteins could be at the base of a wide range of physiological responses of different muscle fibers, allowing a rapid and fine-tuned adaptation to the changes in contractile demands.

As seen for the AMPD1 isoforms, cardiac and skeletal muscle TnT are proteolyzed in vivo by calpains. During physiological and pathological adaptation of cardiac muscle, such as ischemia–reperfusion and pressure overload, a restricted proteolysis of cardiac TnT by m-calpain occurs that results in the selective removal of the 71 aa N-terminal variable domain not only altering the binding affinities for Tm, TnC and TnI but also lowering the myosin ATPase activity and contractile force generation [80, 81].

Similarly to the cardiac muscle, physio-pathological changes in skeletal muscle induce a compensatory switch for both TnT and AMPD isoforms. In various neuromuscular diseases or after sciatic nerve denervation, the cardiac isoform of TnT, expressed in skeletal muscles only during the fibers development, is up-regulated in the adult fibers [147]. In rodent skeletal muscle atrophy characterized by a preferential loss of fast-twitch fibers, the expression of many enzymes involved in ATP production and glycolysis is down-regulated, whereas other enzymes, including AMPD3 are markedly up-regulated [148]. Furthermore, following denervation of rat skeletal muscle, the total AMPD activity increased by 34% due to the increase in AMPD3 expression that resulted in a change of the AMPD1/AMPD3 subunit ratio in the composition of the holoenzyme [149].

Parnas and Lewinski [90] reported that the amount of NH_3_ developed from frog muscles was not proportional to the amount of muscle work executed since in muscles performing a similar number of contractions the ammonia production increased in the months of activity from January till June but decreased in the months leading to hibernation (November). Taking into account what was observed in the heart of toad where the slow TnT1 isoform replaces cardiac TnT2, suggesting a physiological value to this evolutionary adaptation by which the toad heart is able more than the frog heart to cope with the decrease of blood volume that occurs during dry season, it would be interesting to determine whether the increase in NH_3_ production is linked to the expression of different isoforms of AMPD or the regulation of the AMPD activity changes as a physiological adaptation to obtain a higher capacity of fast contractions required for example for jumping.

The above-described similarities between TnT and AMPD such as the isoform switch during muscle development, the adaptation to different contractile necessities or the readjusting to the change of environmental conditions, the restricted proteolysis by calpains of the N-terminal domains containing both a putative zinc-binding site and the nuclear localization shared by the two proteins, altogether suggest a distinctive relationship between TnT and AMPD. The ability of the TnT variable N-terminal region to interact through a zinc bridge with the enzyme catalyzing the deamination of AMP to maintain a high energy charge and supply myosin ATPase with ATP could be at the base of a rapid and fine-tuned adaptation to the changes in contractile demands.

In this context also deserves consideration the observation that in muscle the cytoplasmic free zinc concentration is very low (about 10^–10^ M) [150] suggesting that the presence of intracellular Zn-chaperones could be critical for the physiological functions of a Zn metalloenzyme such as AMPD and sarcomeric proteins that have zinc-binding sites such as TnT. This is particularly true for the zinc-binding protein HPRG that we have shown to be candidate to provide the zinc ions to stabilize the AMPD1 native conformation.

The zinc dinuclear center present in rabbit fast-twitch AMPD1 could have a regulatory role in holding the enzyme in a less active conformation during muscle contractions under normoxic conditions. However, during strenuous exercise and to the greatest extent during rigor mortis [151], the unrestrained AMPD1 activity caused by the calpain-dependent cleavage of the enzyme N-terminus is responsible for the large ammonia production [12, 13]. Interestingly, based on the comparison of the amino acid sequences, the zinc dinuclear center, formed by the regulatory N-terminal domain and the catalytic C-terminal domain, is probably present in human AMPD1 as well as in the rabbit enzyme but not in rat AMPD1, suggesting that the pH-dependent complex regulation of AMPD1 linked to such structure might have a specific function in human fast skeletal muscle fibers IIA and IIB that have higher shortening velocity but are generally prone to fatigue [152]. In human, the fast-like type II muscle fibers predominate in the hands and in several cutaneous facial (mimetic) muscles, in the tongue and in the larynx (reviewed in [115]). During human evolution, the finger fine movements and the coordination of muscles involved in blinking, mastication, sucking and emitting sounds certainly required a very precise and complex regulation and the fine synergy between slow and fast muscle fibers might have contributed to the development of hand dexterity, thumb prehension, emotional expression and phonation.

## Data Availability

Data supporting this study are included within the article and/or supporting materials.
